# Kinetics of the Solution-Mediated Polymorphic Transformation of the Novel l-Carnitine Orotate Polymorph, Form-II

**DOI:** 10.3390/pharmaceutics10040171

**Published:** 2018-10-01

**Authors:** Ji-Hun An, Wonno Youn, Alice Nguvoko Kiyonga, Changjin Lim, Minho Park, Young-Ger Suh, Hyung Chul Ryu, Jae Sun Kim, Chun-Woong Park, Kiwon Jung

**Affiliations:** 1 Institute of Pharmaceutical Sciences, College of Pharmacy, CHA University, Sungnam 13844, Korea; ajh@chauniv.ac.kr (J.-H.A.); gabriella@chauniv.ac.kr (A.N.K.); koryoi0709@gmail.com (C.L.); minho.park92@gmail.com (M.P); ygsuh@cha.ac.kr (Y.-G.S); 2 College of Pharmacy, Chungbuk National University, Cheongiu 28644, Korea; wonno80@naver.com; 3 R&D Center, J2H Biotech, Suwon 16648, Korea; daman-ryu@j2hbio.com (H.C.R.); jsbach@j2hbio.com (J.S.K.)

**Keywords:** polymorphs, active pharmaceutical ingredient, polymorphic transformation, l-Carnitine orotate

## Abstract

Research studies related to the polymorphs of l-Carnitine orotate (CO), a medication used for the treatment and prevention of liver diseases, are insignificant or almost nonexistent. Accordingly, in the present study, l-Carnitine orotate (CO) was prepared for investigating CO polymorphs. Here, a reactive crystallization was induced by reacting 1g of l-Carn (1 equivalent) and 0.97 g of OA (1 equivalent) in methanol (MeOH); as a result, CO form-I and CO form-II polymorphs were obtained after 1 h and 16 h of stirring, respectively. The characterization of CO polymorphs was carried out utilizing Powder X-ray diffraction (PXRD), Differential Scanning Calorimetry (DSC), Thermogravimetric Analysis (TGA) and solid-state Nuclear Magnetic Resonance Spectroscopy (solid-state CP/MAS ^13^C-NMR). The solution-mediated polymorphic transformation (SMPT) of CO polymorphs was investigated in MeOH at controlled temperature and fixed rotational speed. The results revealed that CO form-I is a metastable polymorph while CO form-II is a stable polymorph. From the same results, it was confirmed that CO form-I was converted to CO form-II during the polymorphic phase transformation process. Moreover, it was assessed that the increase in temperature and supersaturation level significantly promotes the rate of nucleation, as well as the rate of mass transfer of CO form-II. In addition, nucleation and mass transfer equations were employed for the quantitative determination of SMPT experimental results. Lastly, it was suggested that CO form-II was more thermodynamically stable than CO form-I and that both polymorphs belong to the monotropic system.

## 1. Introduction

The solid states (polymorphs, salt, cocrystals, multicomponent crystals, crystallites, co-amorphous, and amorphous) of Active Pharmaceutical Ingredient (APIs) have a significant impact on the solubility and dissolution behavior of these APIs, and therefore alter the drug’s bioavailability. Therefore, several studies focusing on the design and development of methods for controlling the drug’s solid states have been actively conducted, recently [1,2,3,4,5].

In addition, polymorphs are crucial, owing to their influence on the stability of APIs. Thus, the screening and control of APIs’ polymorphs are extremely important. If such studies are not thoroughly conducted, problems can be encountered during the formulation, processing and storage of these APIs [1,2]. The crystallization method for polymorph control has been used to investigate and monitor the transition from a metastable form to a stable form via polymorphic transformation [6,7].

Polymorphic transformation techniques include solid-state polymorphic transformation, in which a solid material undergoes phase transformation to another solid material, and the solution-mediated polymorphic transformation (SMPT), where the phase transformation from a metastable form to a stable form occurs in the solution phase. The SMPT is widely utilized because of its relatively low consumption of energy. Based on Ostwald’s Rule of Stages, the SMPT includes the following 3 steps: the dissolution of the metastable polymorph; the nucleation of the stable polymorph; and the growth of the stable polymorph. Furthermore, by monitoring and controlling the polymorphic transformation in real time, it is possible to promote or lessen—namely, to adjust—the rate of polymorphic transformation [6,7,8,9]. 

Factors influencing the rate of polymorphic transformation include thermodynamic and dynamic factors. Thermodynamic factors illustrate parameters that impact or control the driving force for the polymorphic transformation, namely, the polymorphs’ supersaturation [10,11,12]. This includes temperature, type of solvent, solvent composition, and so on [6,9,13,14,15,16,17,18]. Moreover, factors such as solvent viscosity, rotational speed, concentration of suspension, additive or seed addition, particle size, and so on dynamically affect the polymorphic transformation and physically impact the rate of polymorphic transformation [18,19,20,21].

**Figure 1:**
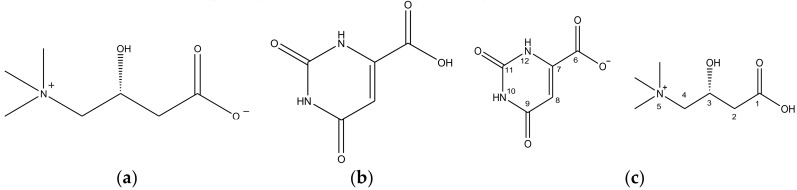
Molecular structure with the atom numbering: (**a**) l-Carnitine (l-Carn), (**b**) Orotic acid (OA). (**c**) l-Carnitine orotate (CO).

Upon administration, l-Carnitine orotate (CO) (Figure 1c) is primarily absorbed in the intestine and thereafter moves to liver tissues. Upon arrival in the hepatic tissues, CO breaks down into l-carnitine (l-Carn (Figure 1a)) and orotic acid (OA (Figure 1b)), and subsequently generates therapeutic effects. CO is presently manufactured and marketed as GODEX capsules or as Ganezin, by Celltrion Pharmaceutical, and it is employed for the treatment of hepatic diseases characterized by an increase of serum Glutamic Pyruvic Transaminase (sGPT) in liver tissues [22,23]. Moreover, CO is available in a crystalline form. In general, most crystalline solids possess more than two polymorphs. The investigation of the polymorphs of crystalline drugs is essential, because polymorphs are key factors in determining the manufacturability, solubility, storage stability, te ease of formulation, and, consequently, the bioavailability of drug materials.

Recently, Kim et al. [23] reported results related to CO polymorph; however, detailed results regarding CO polymorphs were not described in their study. They only reported characteristics of one CO polymorph, which they named form-I. Based on the CO DSC curve reported by Kim et al. [23], we predicted that form-I may undergo polymorphic transformation to another polymorph.

Therefore, in this study, experiments were carried out to investigate polymorphs of CO. A reactive crystallization was induced by reacting 1g of l-Carn (1 equivalent) and 0.97 g of OA (1 equivalent) in methanol (MeOH), ethanol (EtOH), isopropyl alcohol (IPA), ethyl acetate (EA) and methylene chloride (MC), respectively. Vacuum filtration was conducted to collect the crystals. The obtained crystals were analyzed by means of DSC to determine whether CO (Figure 1c) had been synthesized or not. As a result, it was found that crystals obtained from MeOH solvent possess similar DSC curve with CO form-I previously reported by Kim et al. [23] (Figure S1). In addition, after the formation of CO crystals from MeOH, the same mixture was stirred for sixteen more hours. Here, via Powder X-ray diffraction (PXRD), Differential Scanning Calorimetry (DSC), Thermogravimetric Analysis (TGA) and solid-state CP/MAS ^13^C-NMR (SS ^13^C-NMR), it was assessed that a novel polymorph different from CO form-I, reported by Kim et al. [23], was produced. The novel polymorph was named form-II.

Accordingly, the present study aims to report the novel CO polymorph, as well as the characterization results of CO polymorphs using PXRD, DSC, TGA, solution-state NMR and solid-state CP/MAS ^13^C-NMR. The same study also intends to report the solution-mediated polymorphic transformation (SMPT) results of CO polymorphs, at varied temperature conditions and fixed solvent composition and rotational speed. The SMPT quantitative measurements were determined by using the nucleation and mass transfer equations.

## 2. Materials and Methods 

### 2.1. Materials

l-carnitine inner salt (Specific Rotation −28.5° to −31.5°, purity higher than 99%) was purchased from Sigma-Aldrich (Darmstadt, Germany). Orotic acid (purity higher than 99%) was kindly provided by J2H biotech. Co., Ltd. (Suwon, Korea), an active pharmaceutical ingredient manufacturing company. Methanol (MeOH), ethanol (EtOH), isopropyl alcohol (IPA), ethyl acetate (EA), methylene chloride (MC) were purchased from DaeJung Chem. Co. Ltd. (Siheung, Korea).

### 2.2. Reaction Crystallization 

1 g of l-carnitine (1 equivalent) and 0.97 g of orotic acid (1 equivalent) were placed in a 100 mL flask and then dissolved in 15mL of an organic solvent (MeOH, EtOH, IPA, EA, or MC). Here, the dissolution was complete only for MeOH, while suspensions were observed for other solvents. Afterwards, the solution or suspensions were stirred for 30 min at 20 °C and 300 rpm rotational speed in the purpose for inducing a reactive crystallization. As a result, crystals were obtained from MeOH whereas suspensions were observed for other solvents. However, along with the obtained crystals, suspended materials obtained from other solvents were also filtered (vacuum filter) and then analyzed via DSC. The DSC measurement curves revealed a new endothermic peak for solid materials obtained from MeOH and EtOH. However, only the OA endothermic peak was observed for solid materials obtained from other solvents (Figure S1). From the DSC analysis results, we noticed that only the crystals obtained from MeOH were identical to CO form-I, a polymorph previously reported by Kim et al. [23] (Figure S1). Furthermore, a solution-state NMR (1D (^1^H, ^13^C) and 2D) analysis was conducted to assess whether CO had been properly synthesized. In addition, a novel CO polymorph, namely CO form-II, was obtained after CO crystals were stirred for 16 h, at 20 °C and 300 rpm in MeOH solvent. The obtained CO form-I and form-II were assessed and characterized by means of PXRD, DSC, TGA and solid-state CP/MAS ^13^C-NMR. 

### 2.3. Solution-Mediated Polymorphic Transformation (SMPT)

Four samples were prepared by dissolving 1 g of CO form-I in 15 mL MeOH, and then stirred at a fixed rotational speed of 300 rpm and varied temperature conditions, including 20 °C, 30 °C, 40 °C, and 50 °C. Sampling was conducted every hour. The process was performed in triplicate and the obtained products were analyzed using DSC. Each sample was vacuum filtered prior to DSC measurements. The DSC areas of the endothermic peaks were recorded, and afterwards, the calculated mean of each sample was employed in the following equation: area of form-I endothermal peak/area of form-II endothermal peak, in order to measure the SMPT.

### 2.4. Differential Scanning Calorimetry (DSC)

The thermal analysis of CO polymorphs was conducted on a DSC Q20 (TA Instruments, Philadelphia, PA, USA) instrument. The temperature range was set from 40 °C to 220 °C and the heating rate was 10 °C/min.

### 2.5. Thermogravimetric Analysis (TGA)

The thermogravimetric measurements of CO polymorphs were recorded on a TGA Q50 (TA Instruments, Philadelphia, PA, USA) instrument under purified nitrogen, at heating rate of 10 °C /min and over a temperature range from 30 to 400 °C.

### 2.6. Powder X-Ray Diffraction (PXRD)

Powder X-ray diffraction (PXRD) analyses of CO polymorphs were performed on a Powder X-ray diffractometer (Bruker, D8 Advance, Billerica, M.A., USA) using Cu Ka radiation. The voltage was 45kV and the current was 40mA. The divergence and scattering slits were set as 1°, while the receiving slit was 0.2 mm. The experiment was performed at scan rate of 3°/min (0.4 s/0.02°) and 2θ scan, ranged from 5° to 35°.

### 2.7. Solution-State Nuclear Magnetic Resonance Spectroscopy (Solution-State NMR)

1D (^1^H, ^13^C) and 2D (COSY, HMQC, and HMBC) analyses of CO, l-carnitine and orotic acid were achieved on an 800 MHz, High-Resolution NMR Spectrometer (Avance, Bruker, Billerica, M.A., USA) in order to investigate the chemical shift changes occurring within the CO molecule compared with l-carnitine and orotic acid. Samples were dissolves in DMSO-d_6_ prior to analysis.

### 2.8. Solid-State Nuclear Magnetic Resonance Spectroscopy (Solid-State CP/MAS ^13^C-NMR)

Solid-state CP/MAS ^13^C-NMR measurements were recorded on a 500 MHz solid-state NMR (Avance II, Bruker, Billerica, M.A., USA) equipped with cross polarization (CP)/magic angle spinning (MAS) sequence pulse for the characterization of CO form-I and form-II crystals. Experimental conditions per sample were as follows: spinning 5 kHz, pulse delay 10 s, contact time 2 ms and analysis time 24 h. 

### 2.9. High-Performance Liquid Chomatography (HPLC) Method for Measurement of CO Solubility in MeOH 

The solubility of CO polymorphs in MeOH solvent was determined according to the method described by Claudio [24]. The measurements were performed on an Agilent HPLC equipment (Agilent 1260, Santa Clara, CA, USA) and the Column was µ-Bondapak NH_2_, 10 μm, 125 Å (Waters, Milford, M.A., USA). The mobile phase consisted of a mixture of solvent; 0.05 M KH_2_PO_4_: acetonitrile = 35:65 (*v*/*v*). The analysis time was 30 min, the flow rate was 0.5 mL/min, and the detection wavelength 205 nm. 

### 2.10. Attenuated Total Reflectance Fourier Transform Infrared Spectroscopy (ATR FT-IR)

ATR-FTIR spectroscopic analyses of CO polymorphs were performed on PerkinElmer Spectrum 100 FT-IR spectrometer equipped with PerkinElmer Universal ATR Sampling Accessory (Boston, M.A., USA) to investigate and determine the hydrogen bonding of CO polymorphs. Here, the experiments were conducted at a 4000 to 650 cm^−1^ spectral range. The number of scans was 150.

### 2.11. Field Emission Scanning Electron Microscope (FE-SEM)

FE-SEM (Carl Zeiss EDAX, Oberkochen, Germany) analyses were conducted with the aim of monitoring and representing the shape images of CO polymorphs. Here, the range of magnification was set from about 100,000 folds. 

## 3. Results and Discussion

### 3.1. Characterization of CO Polymorphs

**Figure 2:**
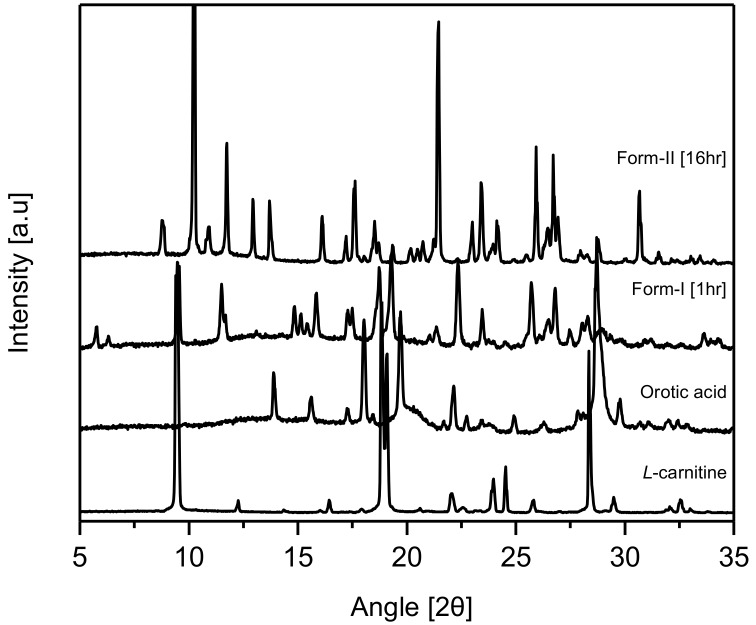
Powder X-ray diffraction (PXRD) patterns of CO.

PXRD experiments were carried out on CO crystals obtained from MeOH after 1 h and 16 h, respectively, for their comparative evaluation with l-Carn and OA. The results revealed that the PXRD patterns of CO crystals were completely different from l-Carn and OA’s ones. Moreover, the PXRD patterns of CO crystals obtained after 1 h coincided with the PXRD patterns of CO form-I, which was previously reported by Kim et al. [23]. However, from the same results, it was confirmed that CO crystals precipitated after 16 h possessed dissimilar PXRD patterns to those precipitated after 1 h. These patterns have not been reported yet in previous studies. Accordingly, it was ascertained that CO crystals collected after 16 h possess a different crystal structure from CO form-I (2 theta: 8.7, 10.3, 11.7, 12.9, 13.7, 16.1, 17.6, 21.5, 23.4, 25.9, 26.7, 30.6). These novel CO crystals were named form-II (2 theta: 9.5, 11.5, 15.8, 18.7, 19.3, 22.3, 25.7, 26.7, 28.7) (Figure 2). As can be seen in Figure 2, the characteristic diffraction peaks for CO form-I appear at 2 theta: 10.3, 21.5, 25.9, 26.7 and the characteristic peaks for CO form-II appear at 2 theta: 9.5, 11.5, 18.7, 19.3, 22.3. 

**Figure 3:**
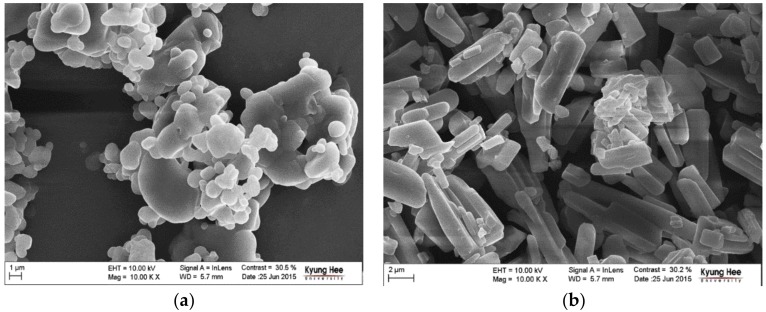
FE-SEM images of CO crystals (**a**) form-I crystals, (**b**) form-II crystals.

Figure 3 depicts the FE-SEM images of CO form-I and CO form-II crystals recorded at 100,000× magnification. The FE-SEM images revealed that CO form-I is condensed and sphere shaped, and CO form-II is long and rectangular shaped. Therefore, it was confirmed that CO form-I and CO form-II were morphologically distinct from each other. Several attempts were made to produce single CO form-I and CO form-II crystals; however, they were unsuccessful at producing good single CO form-I and CO form-II crystals. The non-obtention of single CO form-I crystals is presumably due to the aggregate behavior of CO form-I, which hinders the production of single crystals and leads only to the production of powder materials. However, we were able to obtain long rectangular finely shaped single CO form-II crystals. Here, single crystal X-ray diffraction (SXD) analysis was conducted to determine the crystal structure. However, the obtained transparent crystals changed to opaque crystals during the SXD analysis. Because of this, it was difficult to read the unit cell, and it was consequently hard to predict the crystal structure of CO form-II. Therefore, solid-state CP/MAS 13C NMR measurements, which have been suggested to be efficient analytical techniques for investigating the structural conformation and intermolecular or intermolecular interaction of solid materials [25], were employed in this study for predicting the crystal structures of CO form-I and CO form-II.

**Figure 4:**
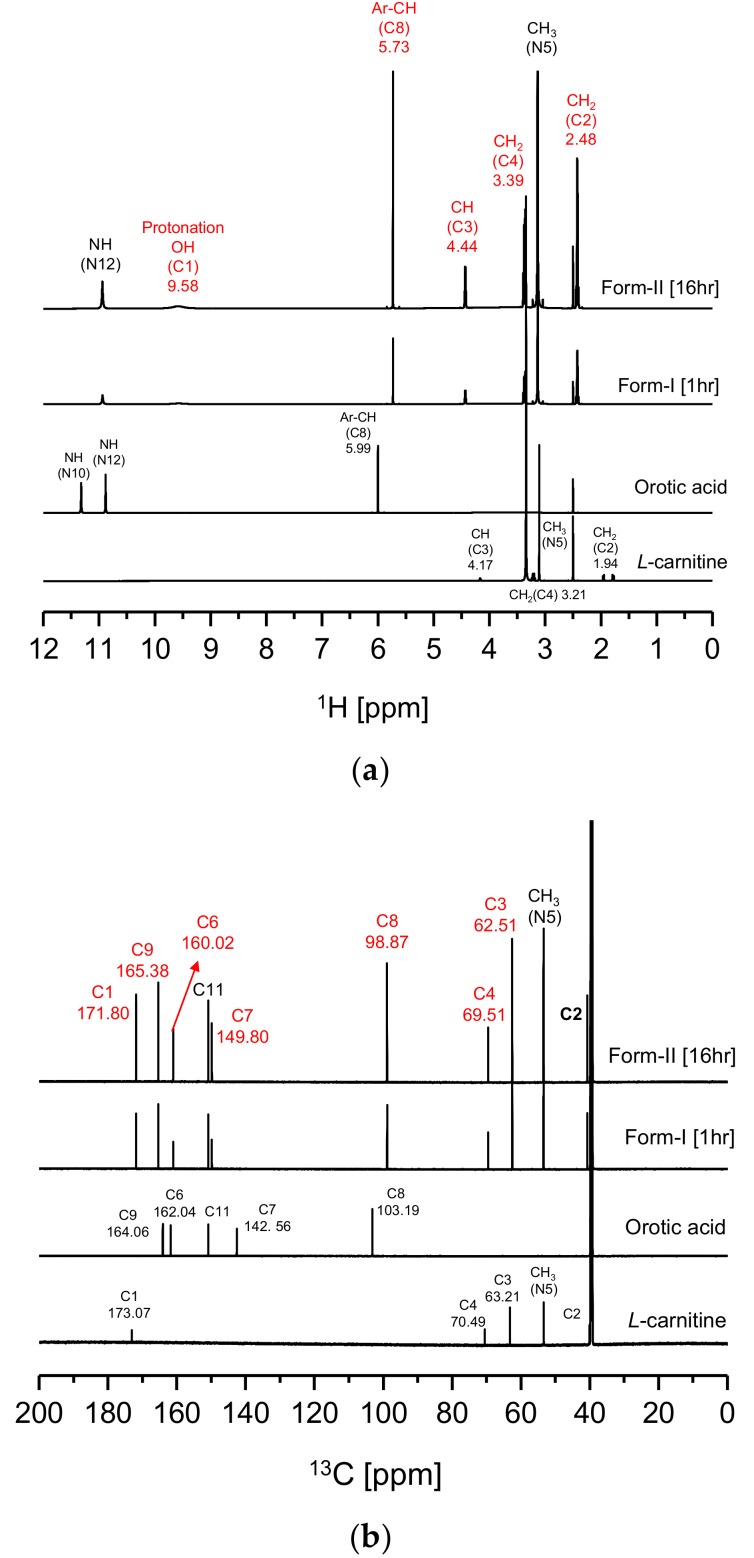
Solution-state 1D-NMR data of CO: (**a**) ^1^H-NMR, (**b**) ^13^C-NMR (numbers on spectral peaks refer to numbers in Figure 1c structure and peak location (DMSO-*d_6_*)).

Figure 4 illustrates the solution-state 1D (^1^H, ^13^C) NMR (DMSO-d_6_) results for CO form-I, form-II, l-Carn and OA. In Figure 4a, a characteristic peak was noticed at 9.58 ppm on the CO form-I and form-II ^1^H-NMR spectra which does not appear on l-Carn and OA ^1^H-NMR spectra. This characteristic peak is presumably the peak of the H atom interacting with the C1’s O atom of CO molecule (Figure 1c). This peak is a result of the migration of the C6 OH’s H atom to C1’s O^-^, resulting in protonation of the C1 region. Moreover, as can be observed from the CO form-I and form-II ^1^H-NMR spectrum in Figure 4a, the peak related to the H of the C8 ring appears upfield at 5.73 ppm than the H peak of O8 (5.99 ppm) in OA spectrum. In addition, in Figure 4b, carbon peaks related to C6 (160.02 ppm) and C8 (98.87 ppm) appear upfield, while peaks related to C7 (149.80 ppm) and C9 (165.38 ppm) appear downfield on the CO’s ^13^C-NMR spectra compared to OA^13^C-NMR spectrum. The result is supposedly induced from the change in the electron density distribution at C6 region due to the deprotonation of C6’s OH. Furthermore, by examining Figure 4a, it can also be noticed that peaks related to C2’s H, C3’s H and C4’s H in the CO molecule in Figure 1c appear downfield, at 2.48 ppm, 3.39 ppm and 4.44 ppm, respectively, on the CO ^1^H-NMR spectra when compared to l-Carn’s spectrum. Furthermore, by comparing the CO and l-Carn’s ^13^C-NMR results, it was found that the peaks of C1 (171.80 ppm), C3 (62.51 ppm), C4 (69.51 ppm) appear upfield on the CO ^13^C-NMR spectrum in comparison to where they appear on l-Carn’s spectrum. This result is a consequence of the change in electron density distribution provoked by the migration of the H from C6’s OH to C1’s O, i.e., due to the protonation of C1 region. From Figure 4, it can be ascertained that CO form-I and form-II possess identical solution-state NMR spectra. This is because both compounds possess the same molecular structure. Based on the solution-state NMR spectra in Figure 4, it was predicted that CO salt would be perfectly produced. Based on CO’s solution-state ^1^H-NMR spectrum integration results, it was confirmed that CO was composed of l-Carn and OA in 1:1 ratio (Figure S2). The solution-state 1D(^1^H, ^13^C) NMR spectra for CO form-I, form-II, l-Carn and OA illustrated in Figure 4 were interpreted based on the solution-state 2D-NMR result, including ^1^H-^1^H COSY, ^1^H-^13^C HSQC, and ^1^H-^13^C HMBC. First, the ^1^H-^1^H COSY results were employed to assign the peaks observed in the ^1^H-NMR spectra in Figure 3a. Afterwards, ^1^H-^13^C HSQC analysis was conducted for the identification of covalent bond peaks made between carbon and hydrogen. Lastly, ^1^H-^13^C HMBC analysis was carried out to determine and assess the peaks of all remaining carbons (Figure S3, Figure S4, Figure S5, Figure S6, Figure S7, Figure S8, Figure S9, Figure S10 and Figure S11).

**Figure 5:**
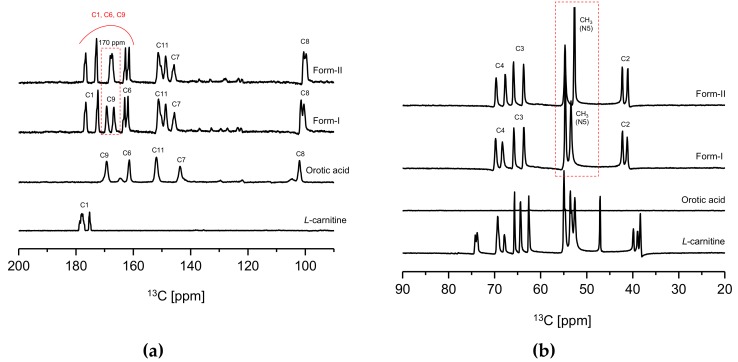
Solid-state CP/MAS ^13^C-NMR data of CO polymorphs: (**a**) 90 ppm–200ppm, (**b**) 20 ppm–90 ppm (numbers on spectral peaks refer to numbers in Figure 1c structure and peak location).

In Figure 5, a significant difference in chemical shifts could be observed from the solid-state CP/MAS ^13^C NMR spectra of CO form-I and form-II, l-Carn and OA. This is presumably due to the differences in the conformation and intermolecular or intermolecular interaction of these compounds [25]. Accordingly, it was predicted that all crystals, namely CO form-I and form-II, l-Carn and OA possess distinct crystal structures. However, in Figure 5b, some difficulties hinder the accurate interpretation of the l-Carn’s solid-state CP/MAS ^13^C NMR spectrum at 20 to 90 ppm region. This is because several peaks on the l-Carn’s solid-state CP/MAS ^13^C NMR spectrum appeared to be split in this region. Hence, in order to provide detailed information about the above-mentioned region, the impact of counter ions on the conformational stability of l-Carn was investigated. Here, l-carnitine HCl salt purchased from Sigma-Aldrich was used for this study and was analyzed via solid-state CP/MAS ^13^C NMR. l-carnitine HCl is a salt in which the orotate anion is replaced by chloride anion. As a result, most CO peaks in the CO spectrum in Figure 4b split into doublets, while most l-carnitine HCl ‘peaks in the l-carnitine HCl spectrum are singlets (Figure S12). This is presumably a result of the differences in chain conformation and orientational order of two salts impacted by the counter ions. However, the salt-free form of carnitine is assumed to be conformationally unstable. Therefore, owing to the conformational instability of l-Carn, solid-state CP/MAS ^13^C NMR data difficult to be accurately interpreted were collected.

Furthermore, a clear difference in carbon peak corresponding to 170 ppm (C1, C6, C9) of the CO molecule (Figure 1c) can be noticed when comparing the CO form-I and form-II’s solid-state CP/MAS ^13^C NMR spectra in Figure 5a. In the solid-state CP/MAS ^13^C NMR spectrum of CO form-I, the 170 ppm peak appears as a doublet; however, the same peak appears as a singlet on CO form-II’s spectrum. Also, when comparing the solid-state CP/MAS 13C NMR spectra in Figure 5b, it can be noticed that the peaks corresponding to CH3 groups, which are bound to the N5 in the CO molecule (Figure 1c), shifted upfield on the form-II spectrum in comparison to the form-I spectrum. The difference in strength of the ionic interaction occurring within molecules in CO polymorphs and consequently the change in conformation of C6’s carboxylate group in Figure 5a is presumed to be the reason; in Figure 5a, the 170 ppm peak appears as a doublet on CO form-I spectrum and as a singlet on CO form-II’s spectrum. The same is assumed to be the reason for the shift of N5’s CH_3_ peak on both CO form-I and CO form-II spectrums in Figure 5b. Moreover, from the same solid-state CP/MAS ^13^C NMR spectra (Figure 5), it can be observed that peaks corresponding to C1, C4, C8 of the CO molecule (Figure 1c) have distinct chemical shifts on both CO form-I and form-II spectra. The result is attributed to the difference in conformation between the two CO polymorphs, which difference is influenced by the intermolecular or intramolecular hydrogen bonding occurring within these two CO polymorphs. Based on the solid-state CP/MAS ^13^C-NMR results in Figure 5, it was confirmed that CO form-I and CO form-II possess dissimilar crystal structures.

**Figure 6:**
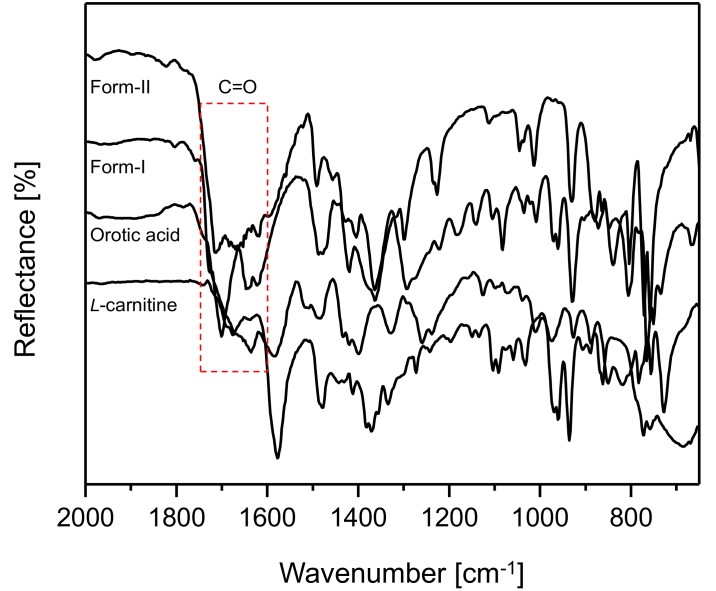
ATR FT-IR spectra for CO form-I and form-II, l-Carn and OA (2000 cm^−1^ to 650 cm^−1^).

Figure 6 illustrates the ATR FT-IR spectral results for CO form-I and CO form-II, l-Carn and OA (2000 cm^−1^ to 650 cm^−1^). The spectral results demonstrated that CO form-I and form-II, as well as l-Carn and OA, possess FT-IR spectra that are distinct from one another. Moreover, CO form-I and CO form-II show a huge difference in the region of the C=O absorption peak, namely in the 1800 cm^−1^ to 1600 cm^−1^ region, when comparing their spectra. This is presumably due to the difference in the strength of the hydrogen boding between molecules in CO form-I and molecules in CO form-II. 

**Figure 7:**
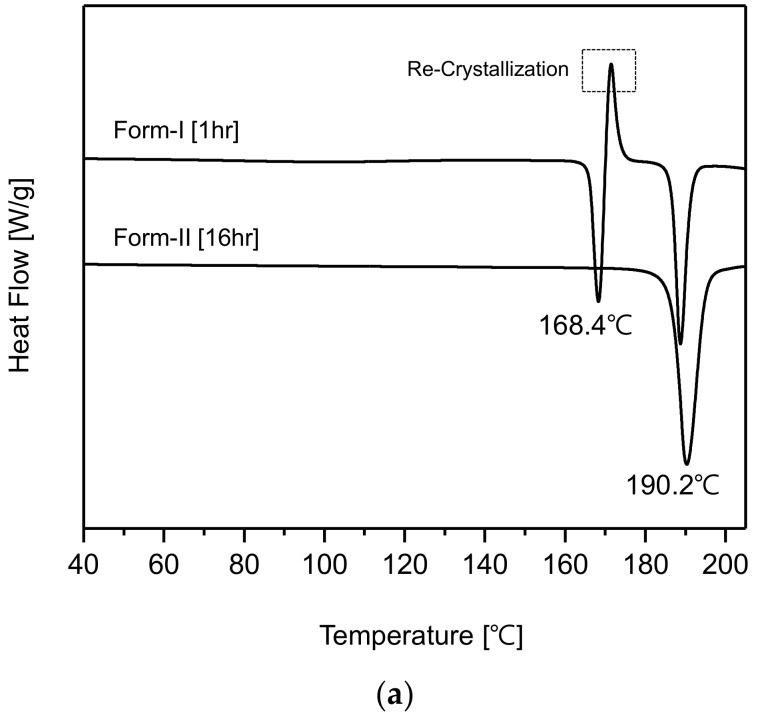

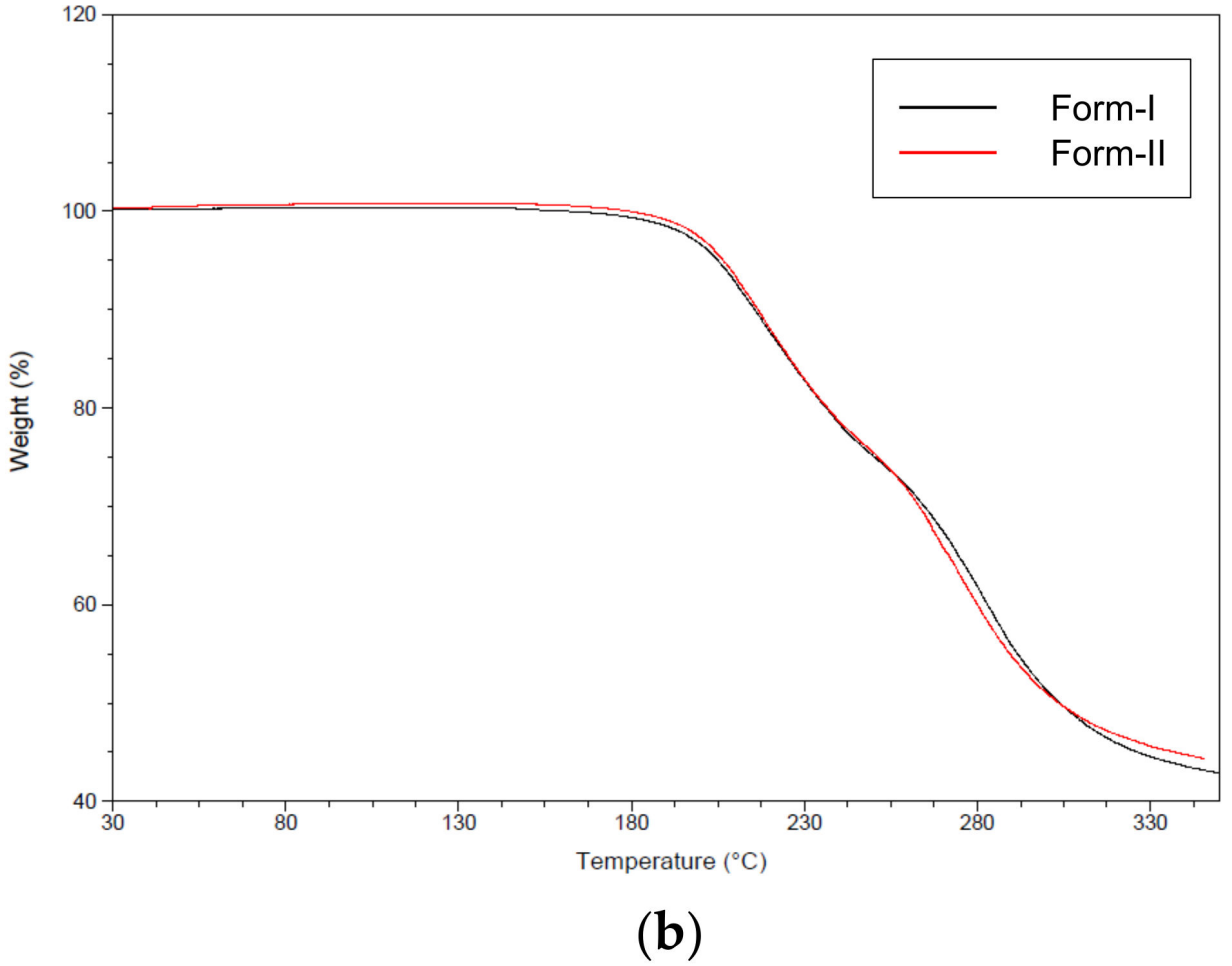
Results of thermal analysis of CO polymorphs (10 °C/min): (**a**) Differential Scanning Calorimetry (DSC), (**b**) Thermogravimetric Analysis (TGA).

Figure 7a represents the DSC curves of CO form-I and form-II polymorphs. The CO form-I’s DSC curve shows an endothermic peak at 168.4 °C and a re-crystallization peak which is followed by another endothermic peak at 190.2 °C. This DSC curve coincides with that of CO form-I reported by Kim et al. [20]. In addition, the DSC curve of form-II shows unique endothermic peak at 190.2 °C. By observing the DSC curves, it was predicted that CO form-I (endothermic peak 168.4 °C) could undergo polymorphic transformation to form-II (endothermic peak 190.2 °C). Accordingly, it was confirmed that CO form-I was metastable polymorph and form-II was stable polymorph. Figure 7b illustrates the TGA analysis results of CO polymorphs form-I and form-II. From this result, no loss in form-I and form-II mass due to the vaporization of solvent was observed. However, as can be seen in the TGA curves, the decomposition of both crystals occurred at approximatively 200 °C. Therefore, it was concluded that both form-I and form-II were ansolvates. 

### 3.2. CO Polymorphs’ Polymorphic Fraction-Dependent Calibration 

**Figure 8:**
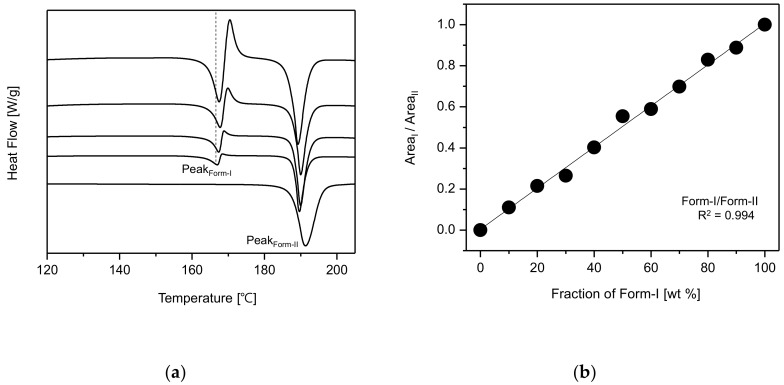
Evaluation of CO form-I and form-II’s polymorphic fractions based on DSC curves: (**a**) change of DSC endothermic peak depending on the polymorphic fraction variation, (**b**) CO polymorphic fraction-dependent DSC endothermic peak calibration.

The experiments for the screening of CO polymorphs were performed under solvent change conditions. The resulting crystals were analyzed using DSC. From the DSC results, it was noticed that the endothermic peak (168.4 °C), as well as the re-crystallization peak, of CO form-I decreased when EtOH was used compared to when MeOH was used (Figure S1). This is presumably due to the presence of both form-I and form-II crystals in the resulting EtOH product. Based on the DSC results illustrated in Figure S1, DSC samples were prepared by varying the fraction (ratio) of form-I and form-II in samples and were afterwards analyzed via DSC. From the DSC results, it was confirmed that both the endothermic peak at 168.4 °C and the re-crystallization peak decreased when the fraction of form-I was inferior than the fraction of form-II in the sample material. However, both peaks increased when the fraction of form-I was superior than that of form-II. Based on these DSC curves, we confirmed the possibility for the quantitative evaluation of CO form-I and form-II polymorphs, depending on their polymorphic fraction (Figure 8a). 

Samples were prepared by mixing form-I and form-II in different ratios, which then were analyzed by DSC for the quantitative determination of the CO polymorphs. The endothermic peak areas were used and calibrated as a function of form-I’s fraction. The calibration was obtained by dividing the endothermic peak area of CO form-I by the endothermic peak area of CO form-II. The R^2^ > 0.994 indicated that the result was trustworthy (Figure 8b). Accordingly, the DSC measurements were conducted based on the results in Figure 6. Afterwards, the DSC endothermic peak areas were employed for the quantitative determination of the recorded CO polymorphs’ SMPT results. 

### 3.3. Solubilities of CO Polymorphs in MeOH for Measuring Their Supersaturation Level 

**Figure 9:**
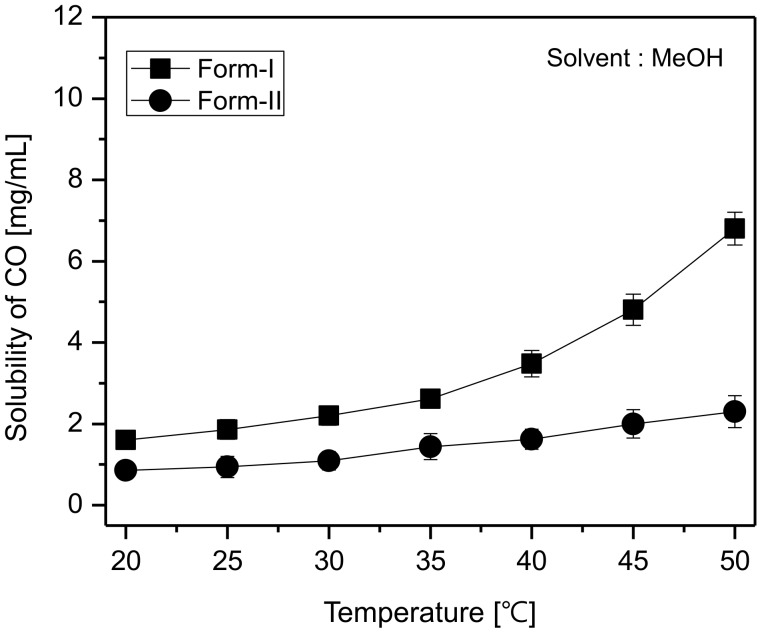
Solubility of CO form-I and form-II in MeOH.

The solubility difference between polymorphs is considered to be the major driving force for inducing the SMPT, it is also considered to be a crucial factor influencing the rate of SMPT. In other words, the supersaturation level (S) impacts the SMPT. The supersaturation can be expressed as S = C*_meta_/C*_stable_. Here, C*_meta_ refers to the equilibrium concentration of the metastable polymorph, and C*_stable_ refers to the equilibrium concentration of the stable polymorph. Therefore, measuring the solubility of the polymorphs is assumed to very important for quantifying SMPT [6,16,18]. Moreover, by measuring the solubility and by plotting the results values, it is possible to predict the polymorphic system of the crystals materials, namely to determine whether the polymorphs are enantiotropic or monotropic. This is because the solubility difference between polymorphs tells us about those polymorphs’ thermodynamic energy [1,2,26]. In this regard, it was assumed that it was critical to determine the MeOH solubilities of CO polymorphs. Here, the solubility of the CO polymorphs was measured according to the HPLC analysis method described above. First, samples of CO form-II were prepared by dissolving a known amount of CO form-II in MeOH at room temperature (25 °C) and then analyzed via HPLC. The recorded peak areas were used for calibration. Afterwards, for the purpose of investigating the effect of temperature on the solubility of CO polymorphs for obtaining their solubility curves, CO form-I and form-II were individually dissolved in MeOH solvent at distinct temperatures set from 20 to 50 °C. Here, CO form-I and form-II were added into the solvent until suspensions were obtained. The mixtures were then stirred for a period of 1 h and thereafter allowed to settle for 1 h. (After suspended materials settled completely, DSC analyses were conducted on CO form-I crystals to verify whether they convert to form-II or not. The DSC results revealed that CO form-I did not convert to form-II after this process). The saturated solutions were separated from undissolved materials using syringes, filtered via a 25 µm membrane filter, diluted 100-fold and subsequently analyzed via HPLC to determine their equilibrium solubilities. The results are illustrated in Figure 9. As can be seen in Figure 9, the solubility difference greatly increases at temperatures above 35 °C. Moreover, from this result, it was predicted that form-I was metastable crystals and form-II was stable crystals. Furthermore, the result demonstrated that the relationship between CO form-I and form-II was monotropic. 

### 3.4. Solution-Mediated Polymorphic Transformation (SMPT) of CO Polymorphs

**Figure 10:**
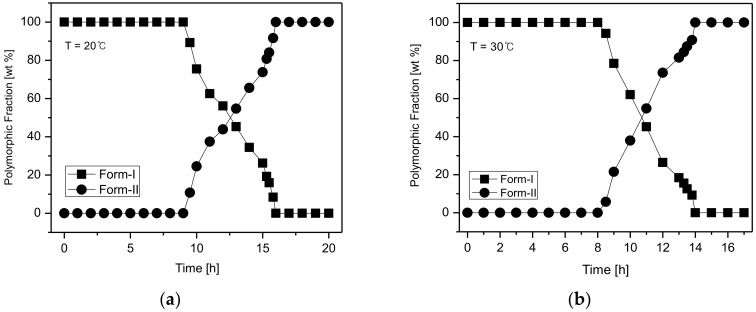

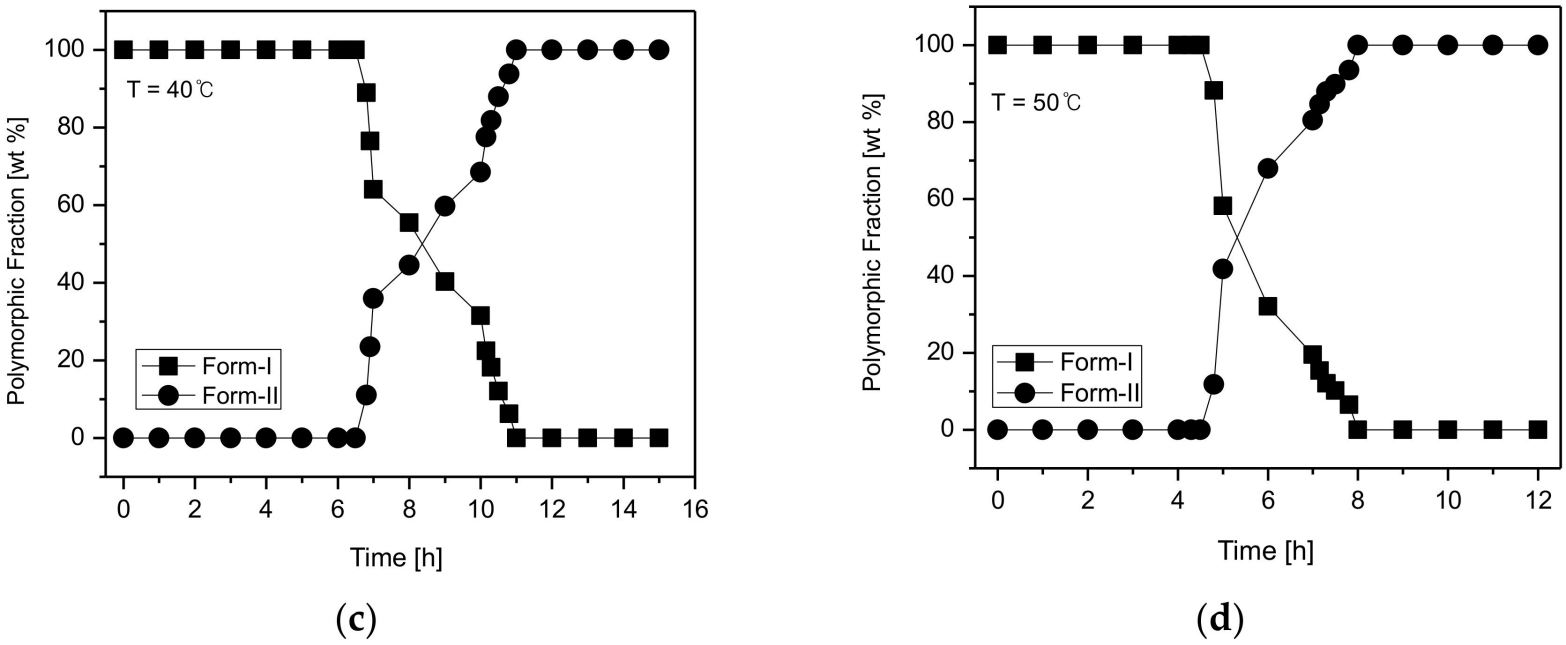
Profiles of the polymorphic fractions variation during the polymorphic transformation process of CO: (**a**) 20 °C, (**b**) 30 °C, (**c**) 40 °C, (**d**) 50 °C.

Figure 10 illustrates the temperature-dependent SMPT profile of CO form-I and form-II. Figure 10a depicts the SMPT profile from CO form-I to CO form-II at 20 °C. The results suggest that at 20 °C, a phase transformation from CO form-I to CO form-II occurs after 15 h. Figure 10b presents the SMPT profile of form-I to form-II at 30 °C. From these results, it was confirmed that CO form-I converts to CO form-II after 14 h. Likewise, the SMPT profile described in Figure 10c revealed that at 40 °C, the phase transformation from CO form-I to form-II occurs after 11 h. Lastly, the SMPT profile in Figure 10d shows the SMPT at 50 °C; CO form-I undergoes polymorphic phase transformation to form-II after 8 h. By observing the SMPT profile provided in Figure 8, it can be predicted that the rate of polymorphic transformation tends to increase with the increase of temperature. Based on the solubility results observed in Figure 9 and the SMPT profiles illustrated in Figure 10, it was ascertained that CO form-I undergoes polymorphic transformation to form-II and two polymorphs are in monotropic relationship [1,2]. 

**Figure 11:**
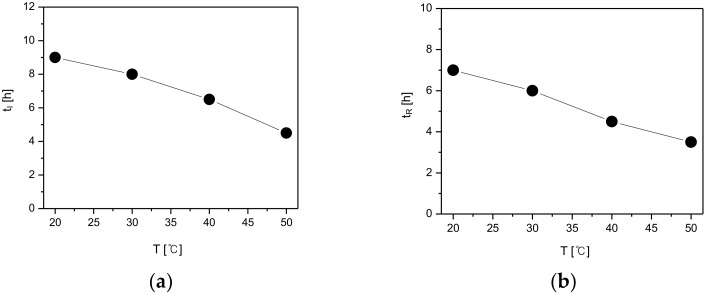
Effect of temperature on polymorphic transformation: (**a**) induction time, and (**b**) reconstruction time.

Generally, the SMPT process includes the simultaneous nucleation of stable crystals and dissolution of the metastable crystals, followed by the growth of stable crystals [9]. Accordingly, for the quantitative determination of the SMPT, the induction time (t_I_) was considered to be the period from the starting time of the SMPT in Figure 8 to the starting time of nucleation, while the reconstruction time (t_R_) was considered to be the interval from the starting time of the phase transformation of the metastable crystals into the stable crystals until completion. The induction and reconstruction times are depicted in Figure 11 based on the SMPT profile of the CO polymorphs illustrated in Figure 10. 

Figure 11a describes the induction time (t_I_) of CO form-II under different temperature conditions. According to the results, the induction time was 9 h when the temperature was 20 °C, 8 h when temperature was 30 °C, 6 h 30min when the temperature was 40 °C, and lastly, 4 h 30 min when the temperature was 50 °C. Additionally, as demonstrated in Figure 11b, with a change in temperature, the reconstruction time from form-I to form-II was 7 h at 20 °C, 6 h at 30 °C, 4 h 30 min at 40 °C, and 3 h 30 min when the temperature was 50 °C. By examining Figure 11, it can be seen that both the induction time and the reconstruction time tend to decrease with the increase of temperature. This is assumed to result from the fact that the increase of temperature improves the solubility difference of CO form-I and form-II (Figure 9), and therefore increases the supersaturation. Here, the increase of supersaturation is assumed to promote form-II’s nucleation and mass transfer rates, and subsequently impact the decrement of both the induction and reconstruction time (Figure 11).

### 3.5. Investigation of Polymorphic Transformation Kinetics of CO Polymorphs 

According to the Volmer’s model, the rate of nucleation depends on temperature and supersaturation. Based on the Volmer’s model, the induction time (t_I_), which is inversely proportional to the nucleation rate, can be expressed in terms of absolute temperature (T) and supersaturation (S) [6,16,18].

(1) $$\;{\ln\;t}_{I}=1/\left[T^{3}\ln^{2}S\right]\;$$

In Equation (1), the supersaturation (S) can be expressed as *S* = C*_meta_/C*_stable_. This equation can be employed to calculate the supersaturation level. Also, as can be seen in Equation (1), the temperature and supersaturation promote the induction time. According to the Volmer’s model, there is a proportional relationship between the rate of nucleation, the temperature (T) and the supersaturation (S) [6,16,18]. In this regard, the effects of temperature and supersaturation on the induction time (t_I_) was quantitatively measured and the results are depicted in Table 1. As demonstrated in Figure 7, CO form-I and form-II’s solubility differences tend to increase with the increase of temperature. Moreover, the supersaturation (S) measurements in Table 1 show that the supersaturation level (*S* = C*_form-I_/C*_form-II_) increase with the increase in temperature. This result can explain the reason for the induction time in Figure 9a has the tendency to decrease when the temperature increases. Here, the increase of temperature is assumed to impact the increase of CO polymorphs supersaturation level, which promotes form-II’s nucleation and consequently provoke the reduction of the induction time. As the induction time is inversely proportional to the nucleation rate [6,16,18]. In the purpose of supporting the predicted result related to the induction time, experiments were carried out using the model equation (Equation (1)) to verify and confirm the accordance between the theoretical values and the experimental values (Table 1). The theoretical results predicted using Equation (1) in Table 1 confirmed that the increase in temperature influences the decrease in the induction time. Moreover, Figure 9a and Table 1 display the experimental results for the induction time, demonstrating that the induction time has the propensity to decrease when the temperature increases. Accordingly, it was assessed that the theoretical results were in good accordance with the experimental results. Based on these results, the decrement of the induction time depending on the increase of temperature is assumed to be a consequence of the improvement of form-II’s nucleation, provoked by the increase of the supersaturation level.

The reconstruction time (*t*_R_) is inversely proportional to the mass transfer rate (*r*) if the total amount of the transformed crystals is consistent. The following expression, *r*~ *k·*Δ*C*, is used when the mass transfer rate (*r*) increases dependent on the transfer coefficient (*k*) and concentration difference (Δ*C*). Here, the concentration difference (Δ*C*) is the difference between the solubility of meta stable polymorph and the stable polymorph (Δ*C* = C*_meta_ − C*_stable_). Moreover, the transfer coefficient (*k*) is in direct proportion to the diffusivity (*D*_AB_). Moreover, according to Stokes–Einstein equation, the diffusivity (*D*_AB_) is directly proportional to temperature (*T*) [6,16]. The Stokes–Einstein equation is as follows.

(2) $$D_{\mathrm{AB}}\;=\;\mathrm{KT}/6{\pi \gamma }_{0\;}\mu \;$$

Therefore, if other parameters are maintained as constants and only the temperature is varied, the relation between reconstruction time (*t*_R_) and mass transfer rate (*r*) using the Stokes–Einstein equation (Equation (2)) can be expressed as below [6,16].

(3) $$\;t_{R}\;\sim \frac{1}{r}\sim 1/\left[T\Delta C\right]\;$$

In Table 2, the effect of temperature on the reconstruction time (*t*_R_) behavior was evaluated and predicted using the temperature dependent-solubility data of CO form-I and form-II depicts in Figure 7. As can be seen in Table 2, the concentration difference (Δ*C*) between form-I and form-II increases with the increase of temperature. Also, the experimental measurements show that the reconstruction time (*t*_R_) decreases with the increase of temperature. The Equation (3) demonstrates that the reconstruction time (*t*_R_) is inversely proportional to the temperature (T) and concentration difference (Δ*C*). Moreover, in Table 2, the theoretical measurements for the reconstruction time, predicted via Equation (3), revealed that the reconstruction time (*t*_R_) tends to decrease with the increase of temperature. Accordingly, the experimental results for reconstruction time illustrated in Figure 9b and Table 2 were assessed to agree with the theoretical results. Here, the decrement in the reconstruction time is presumably due to the increase in temperature, the increase of which presumably promotes the solubility difference between CO form-I and form-II, and therefore improves the rate of mass transfer. 

**Figure 12:**
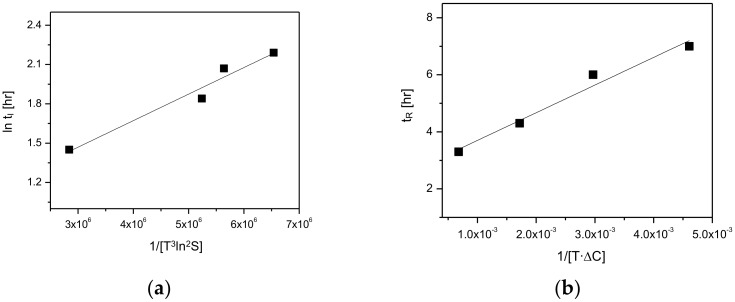
Kinetic correlation of (**a**) the induction time of the polymorphs in phase transformation based on Volmer’s nucleation model, (**b**) reconstruction time of the polymorphs in phase transformation based on the film theory mass of transfer rate.

As depicted in Figure 12, the kinetics of the polymorphic transformation, namely induction and reconstruction time, were plotted as a function of $T^{3}\ln^{2}S$ and/ or TΔC. Figure 12a demonstrates the plotting results for the induction time (*t*_I_) and 1/[$T^{3}\ln^{2}S$] data (Table 1). Here, the result shows a linear dependence of induction time (*t*_I_) and 1/[$T^{3}\ln^{2}S$]. Furthermore, Figure 12b describes the plotting results for the reconstruction time (*t*_R_) and 1/[TΔC] data depicted in Table 2. Based on this result, it was confirmed that the reconstruction time is linearly dependent on 1/[TΔC]. Accordingly, it was ascertained that the kinetics of the polymorphic transformation was significantly influenced by the rate of nucleation and rate of mass transfer. 

The findings of this work are assumed to be essential information to be employed for the selection and production of a desired CO polymorph in term of satisfactory formulation, manufacturing, and storage of CO drug products.

## 4. Conclusions

As an excellent API for treating and preventing liver diseases, CO has been manufactured and sold in a crystalline form for a long period of time. However, studies regarding CO polymorphs are as yet inconsequential. Recently, Kim et al. [23] reported the CO form-I polymorph and its characteristics. However, the result provided by Kim et al. [23] is presumed to be a simple analysis of form-I crystals. Nevertheless, studies involving the screening of CO polymorphs and their polymorphic transformation are still insignificant. Therefore, in this work, two CO polymorphs, including CO form-I, a CO polymorph previously reported by Kim et al. [23], as well a novel CO polymorph, namely, CO form-II, have been reported. Moreover, this study reports an exhaustive characterization of the obtained CO polymorphs carried out using PXRD, DSC, TGA, solution-state NMR (1D(^1^H, ^13^C) and 2D), Solid-State CP/MAS ^13^C-NMR. Moreover, the SMPT of CO form-I and CO form-II was investigated in MeOH solvent at different temperatures. As a result, it was assessed that CO form-I undergoes polymorphic transformation into CO form-II. Additionally, by comparing the solubilities of these two polymorphs in MeOH at distinct temperatures, it was assessed that the polymorphic system for CO form-I and CO form-II belongs to the monotropic category. Moreover, it was confirmed that the induction time for form-II, as well as the reconstruction time from form-I to form-II, decreases with the increment of temperature. The reason for this is that the increase of temperature boosts the supersaturation level (*S* = C*_form-I_/C*_form-II_) of form-I and form-II, as well as the difference of their solubilities (Δ*C* = C*_form-I_ − C*_form-II_), promotes the rates of nucleation and mass transfer, and consequently provokes the decrease of the induction time and reconstruction time. Lastly, with respect to their thermodynamic stability, CO form-II was assessed to be more stable than CO form-I. These results were confirmed by comparing the experimental with the theoretical measurements. In this regard, the findings of this work are assumed to be valuable basic data that are essential for the selection of preferred CO polymorphs suitable for the formulation, manufacturing and storage of CO.

## Figures and Tables

**Table 1:** Correlation of induction time with respect to parameters of the nucleation model.

Form-II			
*T* (°C)	*S*	(*T*^3^ln^2^*S*)^−1^ × 10^−7^	*t*_I_ (h)
20	1.86	6.54	9
30	2.02	5.64	8
40	2.15	5.24	6.3
50	2.96	2.87	4.3

**Table 2:** Correlation of reconstruction time with respect to parameters of mass transfer model.

Form-I to Form-II			
*T* (°C)	Δ*C*	(*T*Δ*C*)^−1^ × 10^−3^	*t*_R_ (h)
20	0.74	4.61	7
30	1.11	2.97	6
40	1.86	1.72	4.3
50	4.5	0.68	3.3

## Supplementary Materials

Figure S1. DSC curves of CO crystals obtained under the solvent change condition; Figure S2. Integrated Solution-state 1H-NMR spectrum of CO crystals (DMSO-d6); Figure S3. CO solution-state 2D-NMR HSQC (DMSO-d6); Figure S4. CO solution-state 2D-NMR HMBC spectrum (DMSO-d6); Figure S5. CO solution-state 2D-NMR COSY spectrum (DMSO-d6); Figure S6. l-carnitine’s solution-state 2D-NMR HSQC spectrum (DMSO-d6); Figure S7. l-carnitine’s solution-state 2D-NMR HMBC (DMSO-d6); Figure S8. l-carnitine’s solution-state 2D-NMR COSY spectrum (DMSO-d6); Figure S9. Solution-state 2D-NMR HSQC spectrum of Orotic acid (DMSO-d6); Figure S10. Orotic acid’s solution-state 2D-NMR HMBC spectrum (DMSO-d6); Figure S11. Orotic acid’s solution-state 2D-NMR COSY spectrum (DMSO-d6); Figure S12. l-Carnitine HCl’s solid-state CP/MAS 13C-NMR spectrum.
