# Supplementary material for: Kinetics of the Solution-Mediated Polymorphic Transformation of the Novel l-Carnitine Orotate Polymorph, Form-II
Source: Pharmaceutics. 2018 Oct 1;10(4):171. doi: 10.3390/pharmaceutics10040171 (PMC6321437; doi:10.3390/pharmaceutics10040171)
Supplement: Supplementary file 1 [file pharmaceutics-10-00171-s001.pdf]

pharmaceutics

MDPI

Supplementary Materials: Kinetics of the Solution-Mediated Polymorphic Transformation of the Novel L-Carnitine Orotate Polymorph, form-II

Ji-Hun An, Wonno Youn, Alice Nguvoko Kiyonga, Changjin Lim, Minho Park, Young-Ger Suh, Hyung Chul Ryu, Jae Sun Kim, Chun-Woong Park and Kiwon Jung

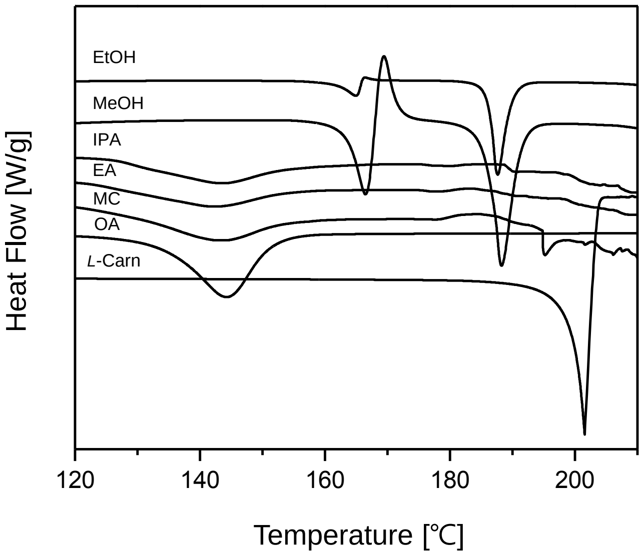

Figure S1. DSC curves of CO crystals obtained under the solvent change condition.

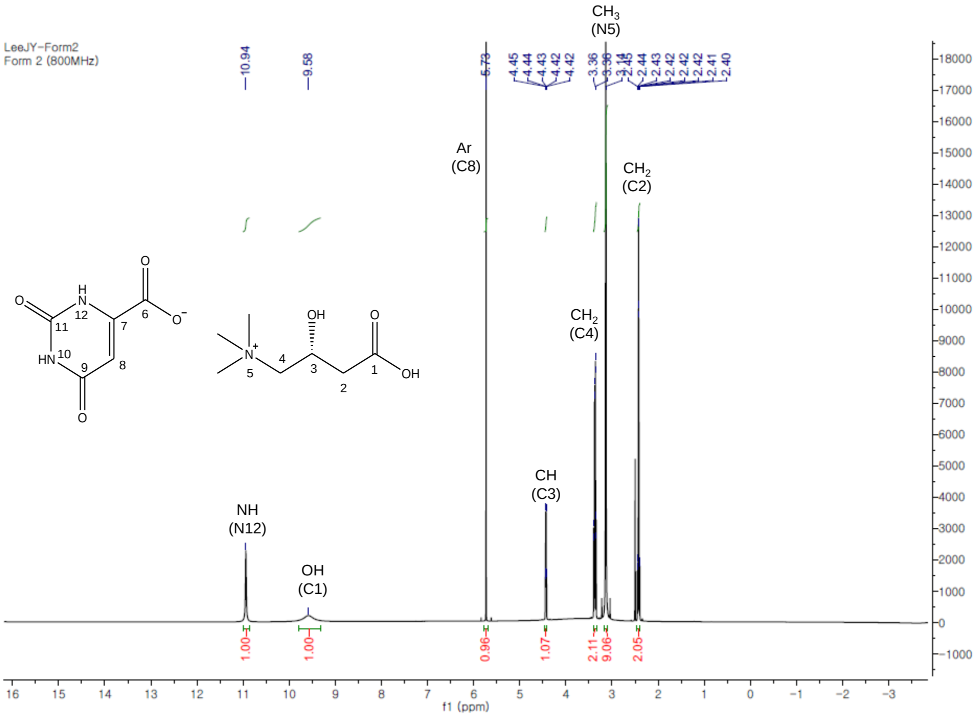

Figure S2. Integrated Solution-state ^{1}H-NMR spectrum of CO crystals (DMSO-d_6).

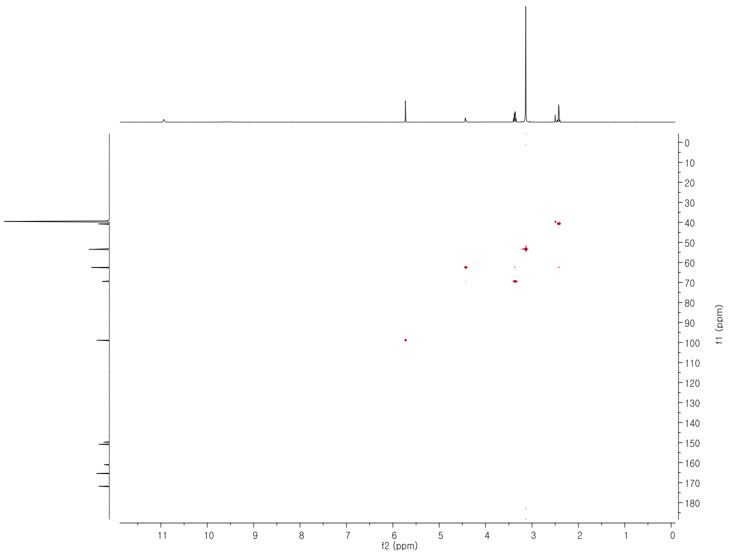

Figure S3. CO solution-state 2D-NMR HSQC (DMSO-d6).

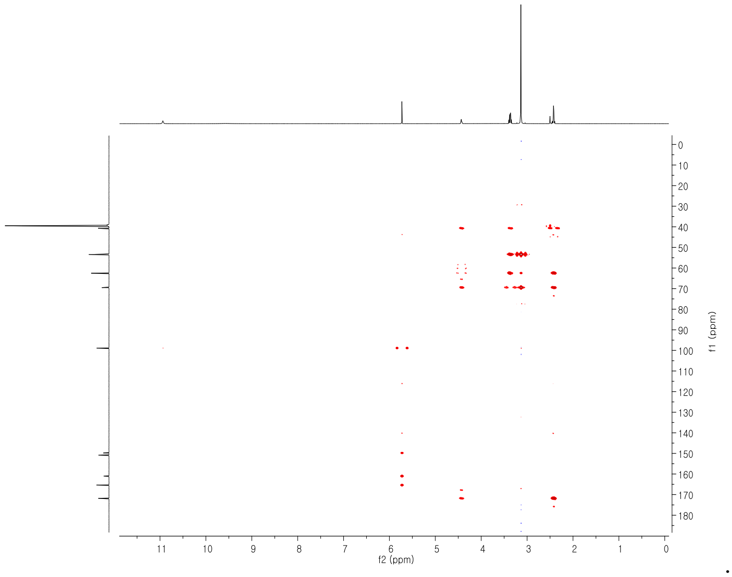

Figure S4. CO solution-state 2D-NMR HMBC spectrum (DMSO-d6).

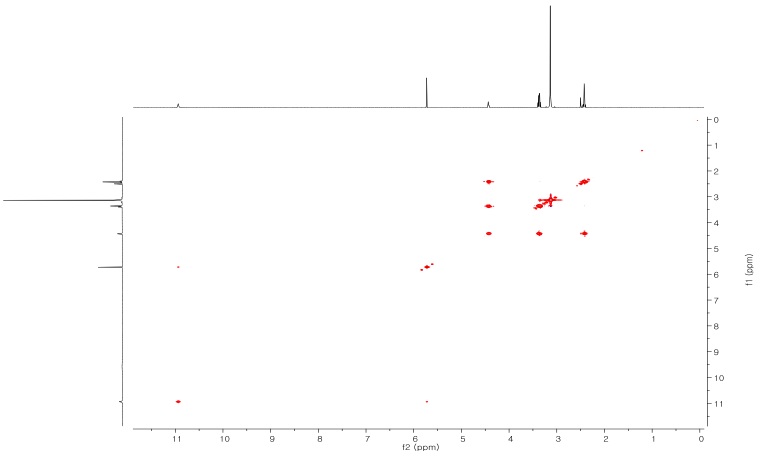

Figure S5. CO solution-state 2D-NMR COSY spectrum (DMSO-d6).

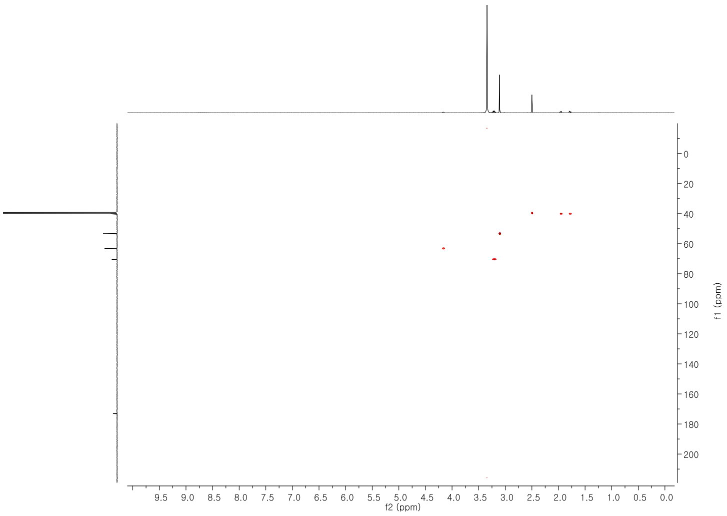

Figure S6. *L*-carnitine's solution-state 2D-NMR HSQC spectrum (DMSO-d_6).

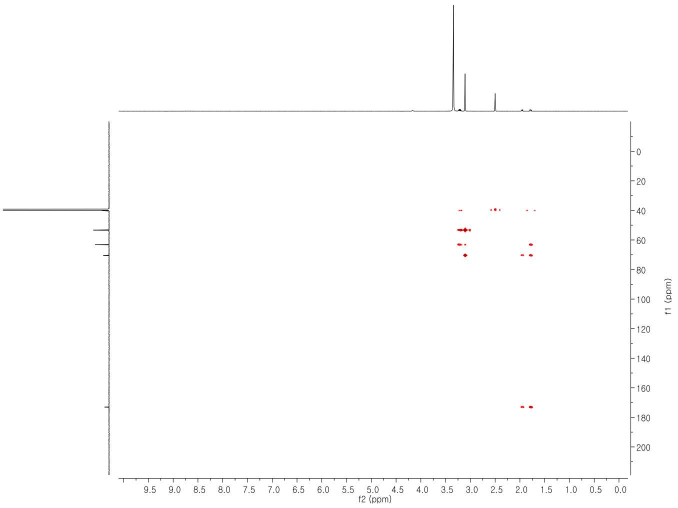

Figure S7. *L*-carnitine's solution-state 2D-NMR HMBC (DMSO-d_6).

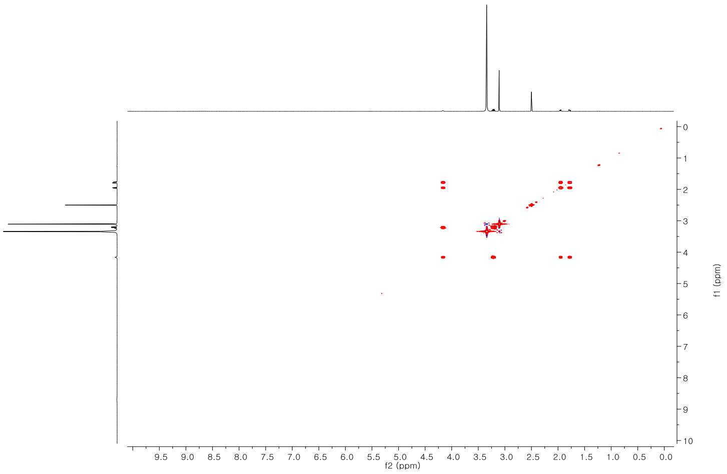

Figure S8. *L*-carnitine's solution-state 2D-NMR COSY spectrum (DMSO-d_6).

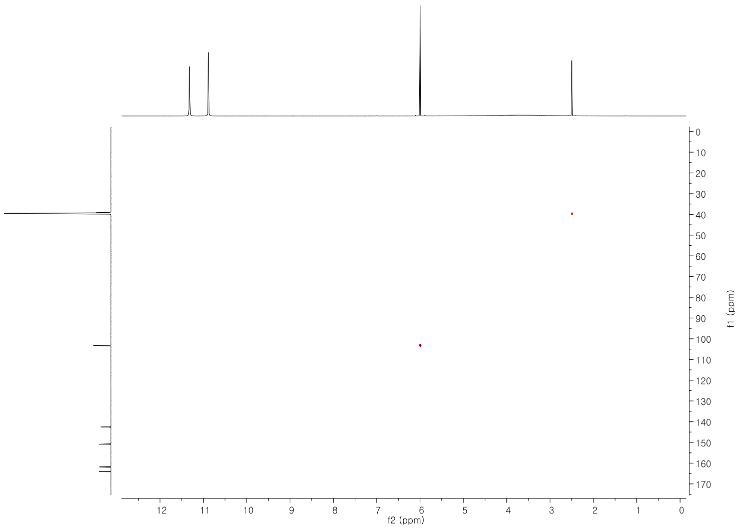

Figure S9. Solution-state 2D-NMR HSQC spectrum of Orotic acid (DMSO-d$_6$).

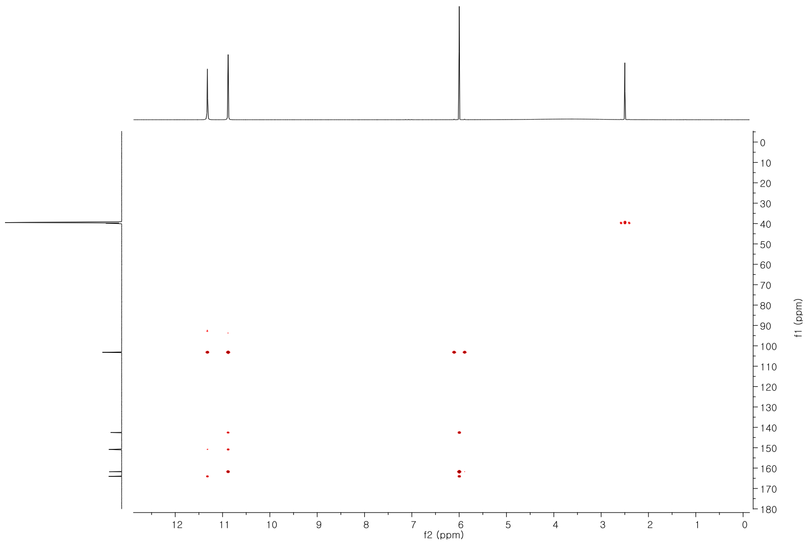

Figure S10. Orotic acid's solution-state 2D-NMR HMBC spectrum (DMSO-d$_6$).

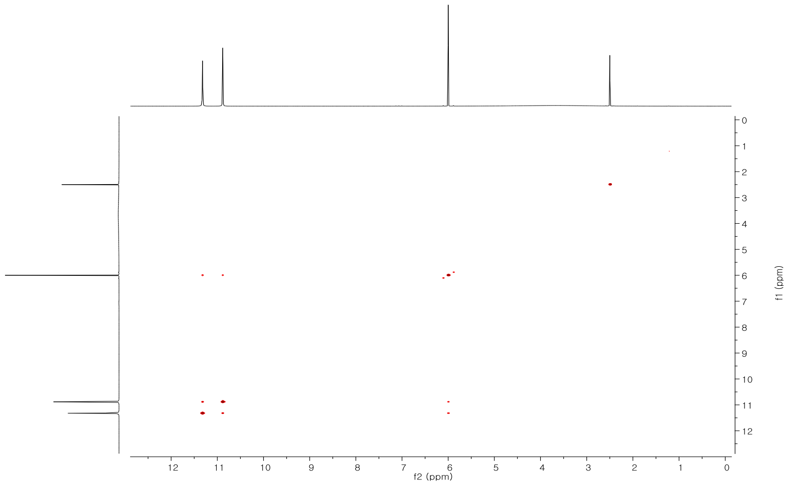

Figure S11. Orotic acid's solution-state 2D-NMR COSY spectrum (DMSO-d$_6$).

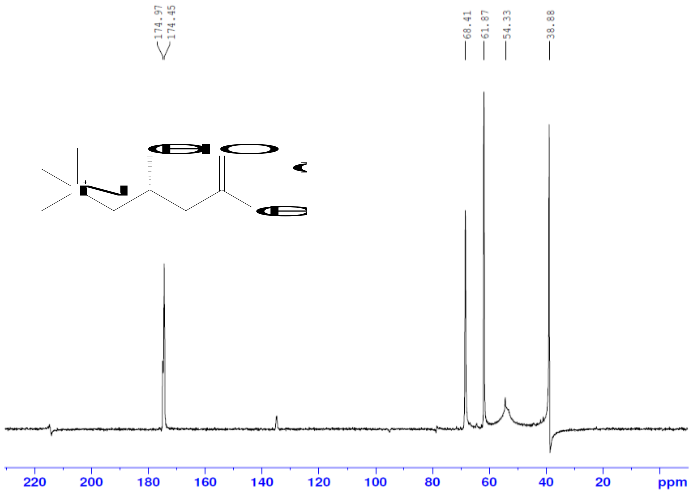

Figure S12. *L*-Carnitine HCl's solid-state CP/MAS ^{13}C-NMR spectrum.
